# Exercise-Induced Improvements to Whole Body Glucose Metabolism in Type 2 Diabetes: The Essential Role of the Liver

**DOI:** 10.3389/fendo.2020.00567

**Published:** 2020-08-28

**Authors:** Shana O. Warner, Michael V. Yao, Rebecca L. Cason, Jason J. Winnick

**Affiliations:** ^1^Division of Endocrinology, Diabetes and Metabolism, Department of Internal Medicine, University of Cincinnati College of Medicine, Cincinnati, OH, United States; ^2^Division of Endocrinology, Cincinnati Children's Hospital Medical Center, Cincinnati, OH, United States

**Keywords:** glucagon, aerobic exercise, resistance training, hepatic glucose production, fasting blood glucose levels, post-prandial glucose

## Abstract

Type 2 diabetes (T2D) is a metabolic disease characterized by obesity, insulin resistance, and the dysfunction of several key glucoregulatory organs. Among these organs, impaired liver function is recognized as one of the earliest contributors to impaired whole-body glucose homeostasis, with well-characterized hepatic insulin resistance resulting in elevated rates of hepatic glucose production (HGP) and fasting hyperglycemia. One portion of this review will provide an overview of how HGP is regulated during the fasted state in healthy humans and how this process becomes dysregulated in patients with T2D. Less well-appreciated is the liver's role in post-prandial glucose metabolism, where it takes up and metabolizes one-third of orally ingested glucose. An abundance of literature has shown that the process of hepatic glucose uptake is impaired in patients with T2D, thereby contributing to glucose intolerance. A second portion of this review will outline how hepatic glucose uptake is regulated during the post-prandial state, and how it becomes dysfunctional in patients with T2D. Finally, it is well-known that exercise training has an insulin-sensitizing effect on the liver, which contributes to improved whole-body glucose metabolism in patients with T2D, thereby making it a cornerstone in the management of the disease. To this end, the impact of exercise on hepatic glucose metabolism will be thoroughly discussed, referencing key findings in the literature. At the same time, sources of heterogeneity that contribute to inconsistent findings in the field will be pointed out, as will important topics for future investigation.

## Introduction

In the United States, type 2 diabetes (T2D) impacts the lives of ~10% of the population (1) by increasing their risk of developing severe health complications associated with micro- and macro-vascular disease. Some risk factors for the development of T2D are non-modifiable, such as age, gender and race (where increasing age, male gender, and Hispanic- and African-American race increase the risk for developing T2D). However, the presence of modifiable risk factors, such as obesity and leading a sedentary lifestyle, can hasten the development of the disease and its debilitating complications. Type 2 diabetes is insidious in nature, but its clinical manifestations are well-known, ranging from elevated plasma insulin and/or slightly elevated glycemia initially (pre-diabetes), to overt diabetes characterized by fasting glucose levels ≥126 mg/dl or glucose levels >199 mg/dl 2 h after a 75-gram oral glucose challenge. While the etiology of T2D is often complex and multi-factorial, it is almost universally characterized by whole-body insulin resistance and the dysregulation of a number of key glucoregulatory organs (2). Among these organs, impaired function of the liver is one of the earliest contributors to impaired blood glucose homeostasis in patients with T2D due to its central role in regulating blood sugar levels during fasting and in response to glucose ingestion. It is for these reasons that it continues to be one of the most thoroughly investigated organs as we develop therapies for T2D.

As recent as the mid-nineteenth century, it was accepted that humans could not make their own metabolic substrates to fuel life. This dogma was finally rejected through the pioneering work of Claude Bernard who, among others, discovered that one of the liver's most important responsibilities is to make glucose and release it into the blood during fasting [for review see reference (3)]. Since that time, the scientific community has worked earnestly to understand the liver's role in the regulation of blood glucose homeostasis, which can vary on a daily basis from eating a meal rich in carbohydrate, where glucose is taken up by the liver and stored as glycogen, to intense exercise, where the liver needs to make glucose at an accelerated rate and release it into the blood to maintain euglycemia. As our knowledge of how the liver responds to such stimuli has greatly expanded over the years, so too has our understanding of the pathology of type 2 diabetes, which is characterized by impaired hepatic regulation of blood glucose homeostasis. Two focal points of this review will be to (1) outline the liver's role in the regulation of blood glucose homeostasis during fasting and feeding and; (2) describe how impaired hepatic responses to these physiological conditions contribute to T2D. Fortunately, ample evidence tells us that exercise can improve hepatic glucose metabolism in patients with T2D, which is undoubtedly part of why it has become a cornerstone of the disease's management. Accordingly, the main emphasis of this review will be to discuss the positive impact that exercise has on liver glucose metabolism in this population. While doing so, studies that directly measured hepatic glucose metabolism (e.g., A-V sampling and isotopic dilution studies) will be highlighted, with a reliance on human subject studies, and periodic references to more mechanistic studies using large and small animal models when necessary.

## Regulation of Hepatic Glucose Production During Fasting

Endogenous glucose production (EGP) includes all of the glucose released into the blood per unit of time, no matter the tissue of origin. In order for any organ to contribute to EGP, two things are required. The first is the capability to enzymatically produce the precursor of glucose, glucose-6-phosphate (G6P), which occurs primarily through two pathways. The most easily accessed source of G6P in the liver is the breakdown of glycogen stores by enzymes (e.g., glycogen phosphorylase and debranching enzyme) through a process called glycogenolysis. The second source of G6P comes from the synthesis of six-carbon G6P by enzymatically combining pairs of three-carbon metabolites (e.g., lactate, glycerol, amino acids, and TCA cycle intermediates) through a process called gluconeogenesis. Once G6P is produced, though, there is no mechanism whereby it can exit most tissues, because it is not a substrate for the glucose transporter proteins (Glut). For this reason, the second requirement to contribute to EGP is the presence of the enzyme glucose-6-phosphatase (G6Pase). G6Pase is found in the endoplasmic reticulum of primarily hepatic and renal cells, and dephosphorylates G6P to make free glucose. This glucose can then move freely out of the cell and into the blood via membrane-indigenous Glut proteins such as Glut-2 (not to be confused with sodium-glucose transporters involved in glucose reabsorption in renal tubules). The liver and kidney are the only two organs that express G6Pase in appreciable amounts, thereby making them the only organs that contribute to EGP. While renal glucose production accounts for ~10% of total EGP, the kidney utilizes as much glucose as it produces, resulting in a net contribution to EGP of zero (4–7). In contrast, the liver accounts for 90% of fasting EGP and undergoes much larger fluctuations in glucose metabolism throughout the course of a day. For example, during high intensity exercise, it is not uncommon for the liver to produce glucose at a rate of 6 mg/kg/min or more to maintain euglycemia in the face of increased metabolic demands of working muscle (8, 9). On the other hand, after the ingestion of carbohydrate, the liver transitions from a glucose production mode, to one that takes up glucose at a rate of ~4–6 mg/kg/min (10, 11). Fasting overnight or in between meals falls near the middle of this metabolic demand continuum, requiring the liver to produce glucose at the modest rate of ~2.2 mg/kg/min so the rest of the body's glucose needs can be met (12, 13). This unparalleled level of metabolic flexibility makes the liver ideally suited to sit at the crossroads of normal blood glucose homeostasis, and why impairments in its function are considered a “canary in the coalmine” for T2D development.

**Figure 1:**
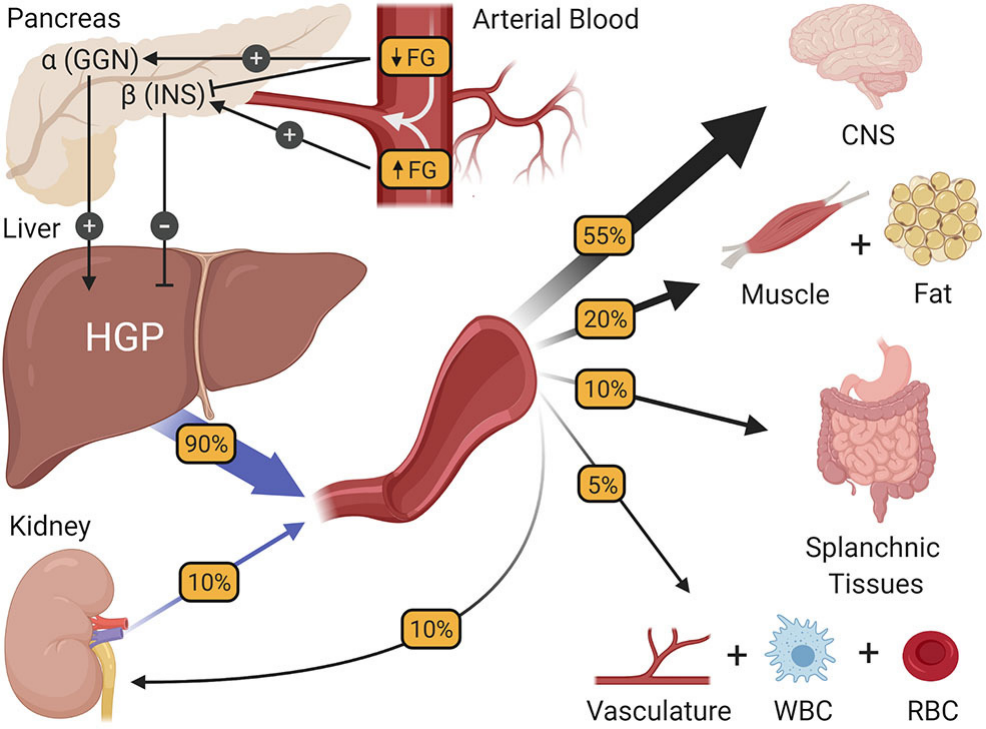
The regulation of fasting blood glucose homeostasis. Subtle changes in fasting glucose levels (FG) entering the pancreas regulate the release of the islet hormones insulin (INS) and glucagon (GGN). In turn, these hormones control the rate of hepatic glucose production (HGP), making HGP equal to the rate at which all other tissues of the body utilize glucose, thereby preserving fasting glucose levels at a steady state. CNS, central nervous system; WBC, white blood cells; RBC, red blood cells.

During periods of modest fasting which, for the purposes of this review refers to periods between normally spaced meals, healthy humans are remarkably adept at maintaining their blood glucose at a very stable level of ~90 mg/dL. While the absence of change may seem unremarkable, it is no small achievement, requiring coordination among multiple glucoregulatory organs. Of the organs purported to regulate fasting blood glucose homeostasis (2), the pancreas and liver interact most closely, precisely matching hepatic glucose production (HGP) with systemic glucose utilization such that euglycemia and normal brain function [which accounts for over one half of fasting glucose utilization (14–17); Figure 1] can be sustained. In healthy humans, this is achieved by subtle, minute to minute changes in the levels of insulin and glucagon at the liver. For example, it is known that when insulin secretion rises in response to a meal, HGP is markedly suppressed, resulting in a conservation of carbon as glucose enters the blood from the small intestine (18). Likewise, increased secretion of glucagon, such as occurs during exercise, can markedly stimulate HGP, preventing a fall in blood glucose as its utilization by skeletal muscle is greatly enhanced (19). While these are extreme challenges to blood glucose homeostasis, their fundamental principles also hold true during the fasting state, when fluctuations in hormones and HGP are less apparent. A good illustration of this comes from the work of Flattem et al. who, in dogs, used a hepatic glycogen phosphorylase inhibitor to modestly reduce endogenous HGP and thereby reduce plasma glucose, or increase glucose entry by intravenous (IV) glucose infusion so as to increase the glucose level (20). The changes in insulin and glucagon that accompanied these fluctuations in blood glucose were carefully assessed by sampling from the animals' hepatic portal vein, which is the vessel that delivers islet hormones to the liver first, followed by their delivery to the rest of the body via venous blood. Sampling from the hepatic portal vein is of particular importance to the accurate measurement of insulin at the liver, because insulin extraction by the liver is ~50–60% in healthy individuals (21), making hepatic portal vein blood insulin levels 2–3 times higher than what is seen by the rest of the body's tissues. Results revealed that the secretion of insulin and glucagon paralleled increases or decreases in plasma glucose levels, respectively, even with a change in glucose of only 10 mg/dL. In fact, the glucose-induced rise in insulin secretion was hardly subtle, with a 50% increase in insulin levels detected in the hepatic portal vein; a change that was undetectable in venous blood (due to hepatic insulin extraction). Notably, an equal fall in glucose inhibited insulin secretion and reduced hepatic portal vein insulin concentrations by ~50%. At the same time, the rise in glucose had little effect on glucagon, but its fall increased glucagon levels in the hepatic portal vein by 50%. These innovative studies sum up perfectly the widely accepted model of how blood glucose homeostasis is normally regulated during fasting; namely that euglycemia is maintained by plasma glucose-induced minute-to-minute changes in insulin and glucagon secretion which, in turn, modify HGP so that it equals the rate of whole-body glucose utilization.

## Dysregulation of Fasting Hepatic Glucose Production in T2D

One criterion for the diagnosis of T2D is a fasting glucose level of 126 mg/dL (7 mmol/L) or higher (22). Previous studies have shown a close association between HGP and elevated fasting glucose, thereby making the former an early contributor to metabolic dysregulation (23). In healthy humans, fasting HGP is ~2.2 mg/kg/min, with one-half of this glucose coming from glycogenolysis and the other half from gluconeogenesis (12, 13). In people who are obese but do not have T2D, chronically elevated insulin secretion allows for the needed suppression of fasting HGP to maintain normal fasting glucose levels, despite the presence of hepatic insulin resistance (24). Predictably, however, as insulin resistance progresses, even hyperinsulinemia is unable to adequately suppress HGP, causing hyperglycemia and T2D. Not to be outdone, hyperglucagonemia is often present in T2D and has also received consideration as a sine qua non of elevated fasting HGP in T2D because of its role in the liver to stimulate HGP and signal in opposition to insulin (25).

Given that hepatic glycogenolysis is more responsive to insulin and glucagon than gluconeogenesis *in vivo* (26, 27), it would stand to reason that an elevation in its rate would be a focal point in the pathology of T2D. Interestingly, however, while some have ascribed the increase in EGP observed in patients with T2D to an increase in G6P production via both metabolic pathways (12), others have observed that increased gluconeogenesis is the primary contributor to elevated EGP (13, 28). The mechanism responsible for increased gluconeogenesis in T2D remains controversial. On the one hand, gluconeogenesis could be enhanced in T2D patients as a result of insulin resistance-induced increases in PEPCK expression. In support of this, Satapati et al. reported that the knockdown of PEPCK, to 40% of normal, protected high-fat fed mice from elevated EGP and the contribution of gluconeogenesis to EGP (29). However, this manipulation of PEPCK also lowered G6Pase expression in the liver, making their relative contributions to the reduction in EGP unclear. On the other hand, Burgess et al. observed that when PEPCK protein was reduced to as little as 30% of normal, gluconeogenesis was not impacted. This led the authors to conclude that the enzyme has a low control coefficient on gluconeogenesis, and that substrate flux is more tightly linked with gluconeogenesis (30). These latter results were further corroborated by Ramnanan et al. who, in dogs, also observed that changes in gluconeogenic substrate flux are not closely tethered to hepatic PEPCK levels, but rather substrate availability (31). In either event, it should be noted that the most proximal cause of elevated HGP is the relative activities of the rate limiting enzymes for HGP; glucokinase (GK) and G6Pase. Because the expression of both enzymes are reciprocally regulated by insulin and glucagon [where insulin increases and decreases GK and G6Pase expression, respectively, and glucagon does the opposite (32)], insulin resistance or hyperglucagonemia can impact the relative activities of these enzymes, favoring the production of glucose over G6P in the liver. Accordingly, this last step of HGP is known to be dysregulated in T2D, thereby leading to excessive HGP (33–35), and making it an important contributor to the etiology of T2D.

A consistent positive energy balance and lack of physical activity are lifestyle factors that track closely with the rise in obesity and T2D, resulting in ectopic fat accumulation and insulin resistance in tissues such as skeletal muscle and liver. Non-alcoholic fatty liver disease (NAFLD) is a common chronic condition that can be caused by excess fat accumulation in the liver (36). NAFLD is a spectrum of diseases, including steatohepatitis which can progress to fibrosis, and potentially more serious liver diseases like cirrhosis and hepatocarcinomas (37, 38), and is estimated to be present in 25–30% of the general population in the United States (39, 40). While NAFLD can be present in normal weight individuals, its prevalence in patients with T2D is closely associated with obesity and increasing BMI (41), with the prevalence among those with T2D being estimated to be as high as 50–60% (37, 41). While consideration of the numerous cellular pathways by which ectopic liver lipid accumulation can contribute to hepatic insulin resistance is beyond the scope of this review, they are thoroughly discussed by Petersen and Shulman (42).

## Impact of Exercise on Fasting Hepatic Glucose Metabolism

Nearly four decades have passed since Bogardus et al. first (to our knowledge) reported that 12 weeks of lifestyle intervention could lower fasting EGP and improve hepatic insulin sensitivity in patients with T2D (43). Since then, considerable effort has gone into improving our understanding of how regular exercise confers its beneficial effects on hepatic glucose metabolism during the fasting state and in response to oral glucose, contributing to the adoption of regular exercise training as a cornerstone in the treatment of T2D.

**Figure 2:**
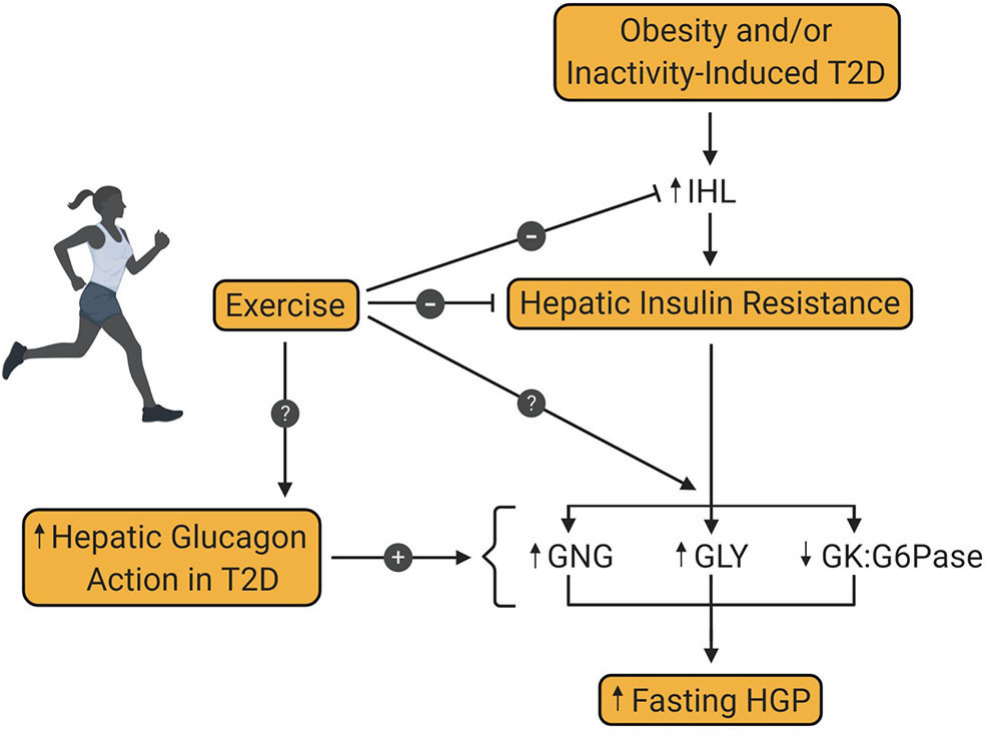
Effect of exercise training on hepatic glucose production in type 2 diabetes. Obesity and/or inactivity are known to be risk factors for the development of type 2 diabetes (T2D). T2D is associated with increased intrahepatic lipid content (IHL) and resultant insulin resistance, which increases fasting hepatic glucose production (HGP). Increased hepatic glucagon action is also a key contributor to excessive HGP in patients with T2D. Exercise training is known to diminish IHL and hepatic insulin resistance in patients with T2D, thereby enhancing insulin mediated suppression of HGP. However, the metabolic pathway(s) impacted by this enhanced suppression is not yet known. Also unknown is the impact of exercise training on glucagon-mediated HGP in patients with T2D. GNG, gluconeogenesis; GLY, glycogenolysis; GK, glucokinase; G6Pase, glucose-6-phosphatase.

In 1984, Bogardus et al. demonstrated the beneficial effect of lifestyle intervention on hepatic glucose metabolism in patients with T2D (43). The results of this study revealed, on the one hand, that exercise-induced weight loss over 12 weeks reduced fasting glucose and insulin, lowered fasting EGP, and improved insulin-mediated suppression of EGP. On the other hand, similar effects on EGP were seen in response to weight loss alone, thereby suggesting that exercise was of no additional benefit to hepatic glucose metabolism. The absence of a no-intervention control group, or a group that performed exercise without weight loss complicated the data interpretation, leaving the question of the independent effect of exercise on hepatic glucose metabolism unanswered. But since then, numerous additional investigations have been conducted, showing the positive impact exercise training *per se*, has on hepatic glucose metabolism, even in the absence of weight loss (Figure 2).

### Hepatic Responses to Aerobic Exercise in People Without T2D

Some of the earlier studies in the field examined the effect of exercise on hepatic glucose metabolism in lean, healthy, non-T2D subjects. In one cross-sectional study, Rodnick et al. (44) observed that healthy, physically trained men had lower rates of EGP at each of two insulin concentrations (10 and 50 μU/ml) compared to untrained counterparts. To the contrary, however, Segal et al. noted that in response to 12 weeks of cycling for 4 h/week at 70% VO_2_ max, fasting EGP was not lowered in lean male control subjects, nor was insulin-induced suppression of EGP enhanced (45). While the 12-week training duration may not have been sufficient to bring about the metabolic benefit that lifetime exercise adherence does, it is also plausible that exercise training in healthy adults would not reasonably be expected to alter liver function that is normal to begin with. In contrast, it is known that hepatic insulin resistance is frequently present in people with obesity (non-T2D). While fasting EGP is not usually elevated in this population, this can be ascribed to increased insulin secretion required to suppress EGP such that hyperglycemia does not occur. Meex et al. (46) showed that 12 weeks of cycling (2x/week for 30 min at 55% of workload max, plus 1x/week of resistance training) by non-T2D subjects with obesity lowered fasting insulin and glucose without impacting EGP, and improved the suppression of EGP in response to hyperinsulinemia. Similar results were published by Shojaee-Moradie et al. (47), who showed that after only 6 weeks of exercise (≥3x/week for 20 min at 60–85% VO_2_ max), insulin-mediated suppression of EGP was improved in a similar population, although no changes in glucose or insulin were seen during fasting. Notably, beneficial alterations in hepatic glucose metabolism as a result of the exercise training in the above-mentioned studies (46, 47) were obtained in the absence of weight loss. In summary, while the effect of exercise training on fasting hepatic glucose metabolism is likely to be negligible in healthy humans, it can clearly be of therapeutic value in people with obesity by improving insulin sensitivity, thereby correcting modest derangements in hepatic glucose metabolism that accompany pre-diabetes.

### Hepatic Responses to Aerobic Exercise in People With T2D

It is also clear that regular aerobic exercise training can improve hepatic glucose metabolism in patients with T2D, although the manifestation of this improvement can vary. In addition to the previously noted results by Bogardus et al. (43), Segal et al. (45) observed that fasting HGP was lower in patients with T2D after 12 weeks of aerobic exercise training (4x/week, 1 h/day, 70% VO_2_ max) without weight loss. On the other hand, most other studies in patients with T2D that used aerobic exercise training durations of 6 weeks or more report that fasting EGP does not change (46, 48–50), even those that incorporate significant weight loss into their study design (48, 49). The reason for this discrepancy is not entirely clear, but this is not to say that subjects with T2D from these latter studies did not demonstrate improved hepatic function as a result of exercise training. In fact, all but one of these studies [which was the only one that showed a lowering of fasting EGP (45)], utilizing an exercise training duration >6 weeks showed that after training, the suppression of EGP by insulin was improved (43, 46, 48–50). Thus, although the manifestations may differ slightly between studies, it is clear that sufficient training durations result in improved hepatic glucose metabolism in patients with T2D, with the most common effect being enhanced suppression of EGP by physiological levels of insulin.

To better understand the nuances about exercise that contribute to improved hepatic glucose metabolism, creative study designs have been employed. For example, it is important to know if the length of a training program is responsible for improved liver function, or if this is a function of the last exercise bout the previous day. Vendelbo et al. (51) did not observe any change in fasting EGP or improved insulin-mediated suppression of EGP 4 h after a single bout of exercise in healthy men (1 h cycling at 65% VO_2_ peak). In contrast, Devlin et al. (52) showed that a single bout of exhaustive, high intensity interval exercise can lower fasting plasma glucose values in subjects with T2D. Moreover, this reduction was accompanied by a 20% decrease in fasting EGP the morning after exercise, as well as enhanced suppression of EGP in response to hyperinsulinemia. While the latter study suggests that improved hepatic glucose metabolism after a single bout of exercise is on par with a 12-week training program, caution should also be applied to the interpretation of this robust response. First, the subjects were not fed after the exercise bout that took place the evening prior, thereby making it possible that the lengthy fast duration after exercise (~12 h) contributed to improved hepatic insulin sensitivity. Second, the exercise bout was exhaustive, which has been shown to reduce the hepatic energy charge in rodents compared to non-exhaustive exercise (53), and most likely had a greater impact on hepatic glucose metabolism than a non-exhaustive bout would have. To our knowledge, these are the only studies that have examined the effect of a single bout of endurance exercise on hepatic glucose metabolism in people with T2D, highlighting the need for future studies to address this question.

Another important question relates to the independent effect of exercise training on hepatic glucose metabolism in patients with T2D. An example of how this can be done, is through the use of a 7-day training protocol, which can improve whole body insulin sensitivity (54), but in the absence of additional insulin-sensitizing metabolic changes that can accompany exercise training (e.g., weight loss). In one such study (55), it was observed that 7-days of aerobic exercise training in patients with T2D (50 min/day at 70% of VO_2_ max) did not lower fasting EGP, nor did it improve insulin mediated suppression of EGP when the expended calories from exercise were replaced with additional food. To the contrary, Kirwan et al. (56) observed that a similar 7-day exercise program (60 min/day at 70% of VO_2_ max), where calories expended during exercise were not replaced, resulted in lower fasting EGP and improved suppression of EGP by insulin. In a study that appears to reconcile these contrasting results, Black et al. (57) observed that in response to 6-days of aerobic exercise consisting of treadmill walking (60–65% of estimated VO_2_ peak for ~60–65 min per day), not replacing the calories expended through exercise (~500 kcal/session) led to improved hepatic function in the form of greater suppression of EGP in response to an IV infusion of glucose, while replacing the expended calories negated this response. To the extent that the different outcomes are regulated similarly (e.g., hyperinsulinemia-induced suppression of EGP and IV glucose-induced suppression of EGP) these studies allow us to infer that in the absence of weight loss and increased aerobic power, a modest, acute energy deficit is required for short term aerobic exercise training to improve hepatic glucose metabolism in patients with T2D.

Consistent with the observation that short-term exercise-induced energy deficits can improve hepatic glucose metabolism in T2D, weight loss can also have a beneficial effect. Bogardus et al. (43) and Coker et al. (49) both showed that weight loss can improve hepatic glucose metabolism in patients with T2D, while Petersen et al. (58) also observed that a body weight loss of 8 kg lowered fasting glucose and insulin concentrations in patients with T2D, in addition to lowering fasting EGP and improving insulin-mediated suppression of EGP. Similar results were presented by Viljanen et al. (59), who showed that a 6-week weight loss program (where 11 kg were lost) in subjects with obesity, lowered fasting EGP. In the latter two studies, it was also observed that hepatic fat content was lowered by 60–80%, thereby giving credence to the hypothesis that lowering liver fat can markedly improve hepatic insulin sensitivity.

As the studies above highlighted, weight loss (58, 59) can significantly reduce intrahepatic lipid content (IHL). Not surprisingly, aerobic exercise training has also been shown to reduce IHL in both patients with T2D and non-diabetic individuals with obesity. A 3 month aerobic exercise intervention (60 min/session at 60–75% VO_2_ peak 3x/week) significantly reduced visceral adipose tissue (VAT) and IHL in adolescents with obesity (60) and 4 weeks of aerobic exercise training (30–45 min/session at 50–70% VO_2_ peak 3x/week) decreased VAT and hepatic TG concentration in men and women who were obese (61). Importantly, changes in IHL content in both studies were achieved in the absence of body weight loss, suggesting an independent effect of exercise in these populations. Likewise, in a study in which patients with T2D and NAFLD were randomized to either an aerobic (60–65% of heart rate reserve) or resistance (70–80% of 1 repetition maximum) exercise training program (3x/week; 60 min/session for 4 months), hepatic fat content was markedly reduced (62). Notably, hepatic fat was decreased comparably with both types of exercise and although dietary intake was not altered and the reduction in BMI was minimal, the decrease in hepatic fat was remarkable (ranging from −26 to −33% from baseline). Given the well-known relationship between IHL and hepatic insulin resistance, the reversal of ectopic hepatic fat accumulation is likely to be a key mechanism by which exercise training exerts its therapeutic effect on hepatic glucose metabolism.

### Hepatic Responses to Resistance Training

While the effect of resistance training on peripheral insulin sensitivity has been extensively studied, showing improved insulin action (63–66), much less is known about its effects on the liver. van der Heijden et al. showed that both aerobic (67) and resistance (68) exercise training improves hepatic insulin sensitivity in adolescents with obesity. In the latter study, it was shown that 12 weeks of strength training (2x/week, 1 h/day, progressive training ranging from ~50% to ~80–85% 3RM, 2–3 sets, 8–20 repetitions) increased lean body mass and strength, without having a significant impact on overall body weight, body fat, or peripheral insulin sensitivity (68). Interestingly, the authors did observe improved hepatic glucose metabolism in response to training, in the form of a significant reduction in fasting EGP. Moreover, it was also noted that this reduction was accounted for entirely by reduced glycogenolysis, with rates of fasting gluconeogenesis remaining stable over time. Improvements in hepatic insulin action have also been found in response to progressive resistance training in various older-adult populations. Honka et al. showed that 4 months of resistance training (3x/week, moderate intensity) in elderly women improved the suppression of EGP by 28% under insulin-stimulated conditions, despite no change in hepatic fat content (69). On the other hand, Croymans et al. (70) found in sedentary, young men with obesity, that while 12 weeks of resistance training (3x/week, 1 h/day, moderate intensity) increased lean body mass, relative strength and skeletal muscle insulin sensitivity, there was no improvement in the hepatic insulin resistance index. A difference in methodology could explain the discrepancy in findings as the latter study calculated hepatic insulin resistance from glucose and insulin data taken during the first 30 min of an oral glucose tolerance test (OGTT), while the former employed the isotopic dilution technique to measure EGP. Thus, resistance training appears to be efficacious in improving hepatic glucose metabolism in certain special populations (e.g., obese, elderly, and children). At this time, we are not aware of any studies that have examined its impact on hepatic glucose metabolism in patients with T2D, but there is reason to believe that it could be efficacious. In addition to the previously described study showing that resistance training can reduce IHL in patients with T2D (62), Pereira et al. (71) showed similar results in rodents. In that study, it was shown that a high fat diet, combined with 14 weeks of strength training, significantly reduced hepatic TG content and improved hepatic insulin sensitivity despite no changes in body mass or adiposity. Notably, resistance training also protected these animals from the diet induced increases in lipogenic enzymes such as acetyl-CoA carboxylase and fatty acid synthase. In summary, there have not yet been any studies examining the impact of resistance training on hepatic glucose metabolism in patients with T2D. On the other hand, the evidence that it can benefit other populations warrants future studies on this topic. If it does confer an additional benefit on hepatic glucose metabolism in T2D, resistance training could be used in conjunction with aerobic exercise training and/or different medications to maximize the therapeutic benefit of exercise. Moreover, many individuals with T2D may prefer resistance over aerobic exercise training, especially when obesity is a comorbidity, which could have the added benefit of improving compliance.

### Hepatic Responses to Glucagon After Aerobic Exercise Training

The assessment of how exercise training improves hepatic glucose metabolism in people with T2D almost invariably centers around how processes like EGP are regulated during the fasting state and in response to hyperinsulinemia. However, this assumes that alterations in glucagon action do not change over the same period. In fact, there are only a handful of studies that have looked at the interaction between exercise training and glucagon action in the liver, the majority of which were performed in rodents. In the only study of its kind in humans, Drouin et al. (72) performed a cross sectional study using trained and untrained male subjects. The metabolic studies were initiated with a pancreatic clamp (the use of somatostatin to inhibit endogenous insulin and glucagon secretion), accompanied by IV-delivered hyperinsulinemia (65 ± 12 and 82 ± 11 pmol/L in trained and untrained, respectively; *p* = NS), and the plasma glucose level was maintained at euglycemia by an IV infusion of dextrose (4.9 mmol/L in both groups). After a 2-h equilibration period under these conditions, an infusion of glucagon (1.5 ng/kg/min) was added and metabolic responses were monitored. In response to hyperglucagonemia, EGP was twice as high in trained individuals, leading to a markedly higher plasma glucose level. In follow up studies (73), the same group demonstrated that when perfused with glucagon, the livers of trained rats had greater glucose output compared to those that were untrained. Another important observation was that the nutritional status (fed vs. fasted), and thus liver glycogen availability, made no difference between groups. This suggests that trained rats had greater glucose output in response to glucagon whether it was after a short fast (when glucagon-induced mobilization of liver glycogen is the primary source of HGP) or a more prolonged fast (when liver glycogen is nearly depleted, leaving gluconeogenesis as the predominant pathway). Similar results have been shown to occur in rats in response to a single bout of exercise (74) and, from a mechanistic basis, this increase in hepatic glucagon sensitivity has been ascribed to an increase in the density of glucagon receptors on the liver after training (75). When considering this from the perspective of health and metabolic dysregulation in patients with T2D, the benefit of training-induced sensitization of the liver to glucagon is not obvious. One possibility, as the authors hypothesized, is that this enhances the capability to rapidly stimulate HGP at the outset of exercise, thereby improving performance. Nevertheless, if exercise training has the same glucagon-sensitizing effect in patients with T2D, then it would be antagonistic to the responses to insulin, thereby making gains in insulin action even greater than what is currently believed. Like resistance training, we are not aware of any studies that have examined how regular exercise training impacts hepatic glucagon action in patients with T2D, which is unfortunate given the increasingly prominent recognition that hyperglucagonemia is being given in the causation of fasting hyperglycemia (25).

In summary, because of its importance in the regulation of whole-body glucose metabolism, the study of hepatic responses to insulin sensitizing exercise training should remain a priority in the scientific community. On the one hand, it is clear that regular aerobic exercise training improves hepatic glucose metabolism in patients with T2D, as long as the structure includes sufficient frequency (≥4 days/week), intensity (≥50–60% of VO_2_ max) and duration (≥6 weeks). On the other hand, important questions linger, such as the mechanism of action by which exercise training improves hepatic glucose metabolism (e.g., effects of IHL levels and glucagon action) and the impact that other exercise modalities (e.g., resistance training) have on the liver. In addition, it will be important to identify sources of heterogeneity in hepatic responses to exercise training, as these can potentially be exploited to maximize exercise's therapeutic potential. Currently, factors such as study design (e.g., training duration length) and methodologies (e.g., what hepatic responses are being measured) are the most obvious contributors to response heterogeneity, but underappreciated sources include weight loss and/or acute energy balance status, both of which are capable of improving hepatic insulin sensitivity independent of exercise. In spite of these confounding factors, the evidence that exercise training with or without weight loss can have a positive impact on hepatic glucose metabolism in patients with obesity and T2D is consistent and overwhelming, thereby cementing its recognition as an important contributor to exercise-induced improvements in whole body glucose metabolism.

## Regulation of Hepatic Glucose Metabolism In Response to Glucose Ingestion

**Figure 3:**
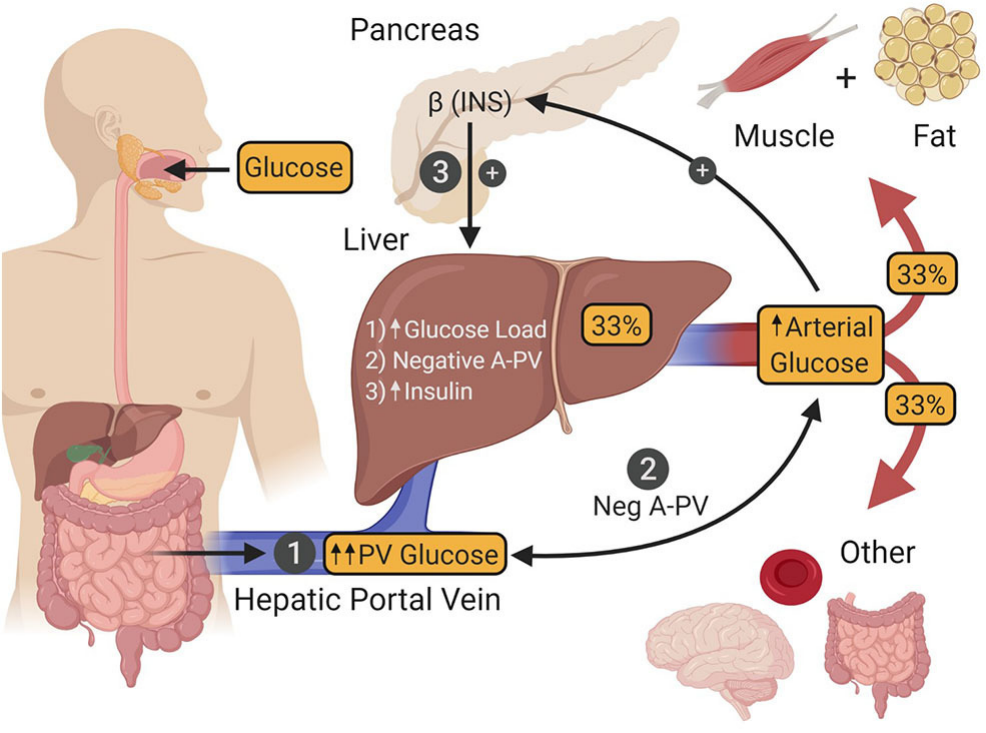
After ingestion, glucose is absorbed into the hepatic portal vein (PV) from the intestine (1). One third of the glucose in the PV is taken up by the liver, after which the remaining fraction is delivered to other tissues of the body by arterial blood. Hepatic glucose uptake is regulated by three factors. The first (1) is an increase in the glucose load to the liver as a result of intestinal glucose absorption-induced hyperglycemia. The second (2) is a negative arterial-PV glucose gradient generated by the absorption of glucose from the intestine into the PV, thereby making its glucose levels higher than that of arterial blood. The third (3) is hyperinsulinemia that occurs as a result of hyperglycemia-induced insulin secretion by islet β-cells. The presence of all three of these signals is required to maximize hepatic glucose uptake. INS, insulin.

During the post-prandial state, which for the purpose of this review means in response to ingestion of a modest glucose load, the liver shifts from producing glucose at a rate of ~2.2 mg/kg/min, to glucose uptake at a rate of ~4–6 mg/kg/min, with ~70% of this being stored as glycogen for later use during fasting (76–78). This shift is accomplished by the interaction of hyperinsulinemia with a pair of important metabolic cues from incoming glucose which, when present at the same time, maximize hepatic glucose uptake, and glycogen synthesis (Figure 3).

Under euglycemic clamp conditions (95 mg/dL), raising the plasma insulin level to 125 μU/mL via an intravenous (IV) infusion of the hormone, leads to a marked increase in skeletal muscle glucose uptake (accounting for ~85% of whole-body glucose utilization) and very little glucose uptake by the liver [~5% (79)]. However, under these metabolic conditions, hyperinsulinemia is the only significant post-prandial metabolic cue present, while important glycemic changes that accompany glucose ingestion are absent (80). One such glycemic cue is hyperglycemia *per se*, which increases the glucose load to the liver, resulting in greater transport of glucose into hepatocytes. While hyperglycemia and hyperinsulinemia are each capable of suppressing HGP, neither can stimulate liver glucose uptake by itself (11, 81). On the other hand, when both are present simultaneously, they are able to stimulate net hepatic glucose uptake to a limited extent [~2 mg/kg/min (11, 82)]. The second glycemic cue required to maximize post-prandial rates of hepatic glucose uptake is a negative glucose gradient between the arterial blood and that of the hepatic portal vein (83, 84) which occurs when glucose is being absorbed into the body from the intestine. This neurally mediated “feeding signal” works synergistically with hyperglycemia and hyperinsulinemia such that hepatic glucose uptake reaches a post-prandial rate of ~4–6 mg/kg/min [Figure 3; (10, 11)]. By juxtaposing this rate of hepatic glucose uptake with the suppression of the pre-meal HGP (~2.2 mg/kg/min), the net change in hepatic glucose metabolism in response to glucose ingestion is actually ~6–8 mg/kg/min, putting it on par with skeletal muscle, where each organ is responsible for taking up approximately one-third of ingested glucose (85–92).

At the cellular level, glucokinase (GK) is the rate limiting enzyme for hepatic glucose uptake and glycogen synthesis. Given its high Km for glucose (8–10 mM) compared to muscle hexokinase-1 (0.1 mM), GK is ideally suited to respond to physiological changes in the plasma glucose level (93). Compared to the concentration dependent inward diffusion of glucose through Glut2 in hepatocytes, the activation of GK is much more complex. GK expression is directly regulated by insulin, thereby making protein levels their lowest toward the end of a fasting period (93), with the remaining GK bound to its regulatory protein (GKRP) in the nucleus of hepatocytes (94). This sequestration allows G6Pase activity and the production of glucose to proceed unhindered between meals so euglycemia can be preserved. However, when glucose is being absorbed from the small intestine after a meal, the transition of the liver to glucose uptake is facilitated by an insulin-induced and neurally triggered increase in GK expression, and a release of existing GK from GKRP in the nucleus, allowing it to translocate out to the cytosol where it can phosphorylate incoming glucose (95, 96). Once the incoming glucose is phosphorylated, ~70% is stored as glycogen for later use during fasting and the remaining fraction proceeds through the glycolytic pathway and is either oxidized (~10%) or released as lactate (~20%) (76–78).

## Dysregulation of Post-Prandial Hepatic Glucose Metabolism In T2D

**Figure 4:**
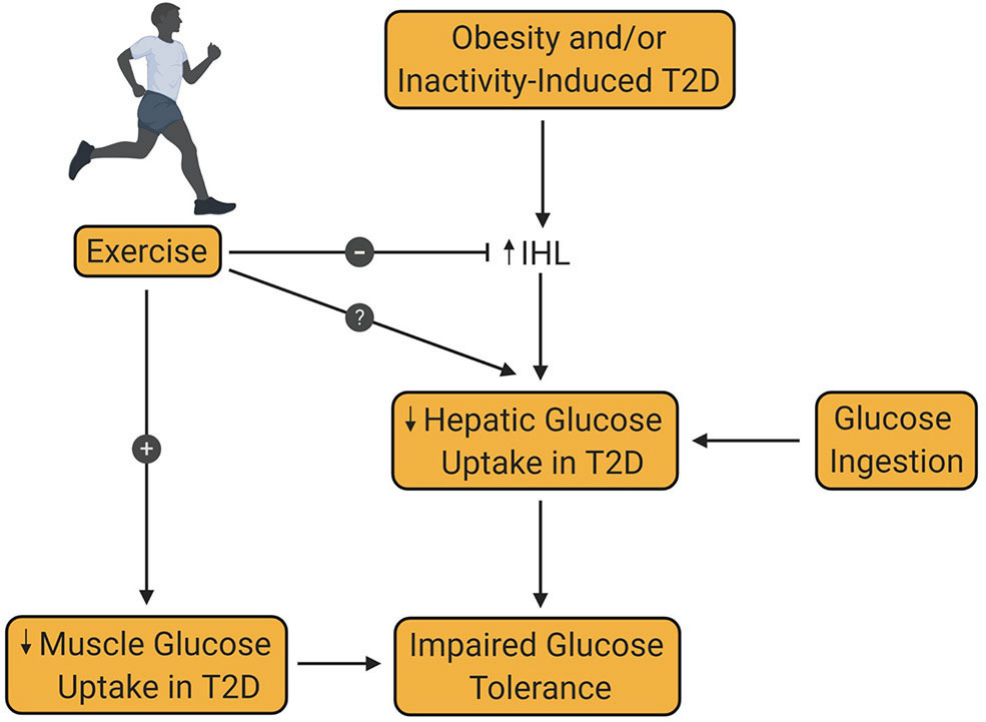
Effect of exercise training on hepatic glucose uptake in type 2 diabetes. Obesity and/or inactivity are known to be risk factors for the development of type 2 diabetes (T2D). T2D is associated with increased intrahepatic lipid content (IHL), which decreases post-prandial hepatic glucose uptake, thereby contributing to glucose intolerance. Decreased muscle glucose uptake in patients with T2D is also an important contributor to glucose intolerance in T2D. Exercise training is known to increase muscle glucose uptake in patients with T2D. At the same time, exercise is known to reduce IHL, although the impact of this reduction on hepatic glucose uptake remains controversial and requires further investigation.

A second criterion for the diagnosis of T2D is a plasma glucose level >199 mg/dL 2 h after an oral glucose challenge (22). In mammals, the liver has two sources of blood; the hepatic artery, which accounts for ~20% of total flow, and the hepatic portal vein, which accounts for the remaining 80%. In animal models, such as the dog, net hepatic glucose balance can be easily calculated using the Fick principle. In the human, catheterization of the hepatic portal vein is not permitted for research purposes, leading to an inability to directly measure net hepatic glucose uptake. Instead, arterial—hepatic vein sampling is used to measure net splanchnic glucose balance (NSGB). The primary limitation of this method is that NSGB is influenced by any glucose produced by or taken up by the intestines and spleen. Nevertheless, glucose utilization by these tissues is known to be low and not impacted by insulin. The use of such techniques has led to ample evidence that splanchnic glucose uptake (SGU) and hepatic glycogen synthesis are impaired in patients with T2D [Figure 4; (86, 97–100)].

In a particularly comprehensive study, Krssak et al. recruited patients with obesity and T2D to study their hepatic responses to a mixed meal and hyperglycemic/ hyperinsulinemia (97). It was demonstrated that hepatic glycogen synthesis was 45% lower in patients with T2D compared to lean controls in response to either a mixed meal test or hyperglycemic-hyperinsulinemia. Given that liver glucose uptake and glycogen synthesis correspond well with one another (78), it is not surprising that a number of studies have demonstrated diminished SGU in patients with T2D. In a series of particularly elegant studies, Basu et al. showed that SGU was lower in patients with T2D during hyperglycemic/hyperinsulinemic conditions and in response to intraduodenal glucose infusions compared to non-diabetic controls (99). To more closely examine hepatic glycogen metabolism in these studies, they used a ^14^C-galactose tracer to show that UDP-glucose flux is also lower in patients with T2D, pointing toward diminished GK activity as the cause of reduced SGU (98). While further exploring the mechanistic basis for reductions in SGU associated with T2D, Coate et al. observed that diet induced glucose intolerance impairs net hepatic glucose uptake and lowers liver glycogen synthesis in dogs (101–105). Moreover, the stimulatory effect of the feeding signal generated by a negative arterial-portal vein glucose gradient on net hepatic glucose uptake was completely ablated (102, 105). At the cellular level, this reduction in glucose uptake was associated with diminished GK protein and activity, which is also consistent with significant hepatic insulin resistance (101, 103, 104). These results are in agreement with the previously described human data (98, 99), demonstrating that post-prandial GK activity is impaired in patients with T2D.

## Impact of Exercise on Post-Prandial Hepatic Glucose Metabolism

### Hepatic Responses to Aerobic Exercise in People Without T2D

Compared to our knowledge of how regular exercise training can improve hepatic insulin sensitivity during fasting, relatively little is known about how exercise impacts hepatic glucose metabolism during the post-prandial state. Maehlum et al. (106) used arterial and hepatic vein sampling in healthy young men to examine splanchnic glucose metabolism in response to a 100 gram oral glucose load ingested either 15 min or 14–15 h after a single session of exercise to exhaustion (cycling at 70% VO_2_ max). Results of that study demonstrated that prior exercise, no matter the timing interval, doubled splanchnic glucose output compared to non-exercise controls. Furthermore, the authors also observed that this increase in glucose escape to the periphery accounted for up to 66% of muscle glycogen repletion. Although it is tempting to ascribe the increase in splanchnic glucose output to a reduction in hepatic glucose uptake, alternate explanations (e.g., increased intestinal absorption of glucose or incomplete glucose absorption) prevent a clear interpretation of these data. Similar results were published by Rose et al. (107), who observed that splanchnic glucose output was 30% greater in endurance trained healthy men 30 min after exercise and that this is associated with enhanced whole-body glucose utilization. Together, these data suggest that in healthy people, more of the ingested glucose escapes the splanchnic bed in favor of delivery to peripheral tissues during the post-exercise recovery period.

The disposition of an oral glucose load after exercise was looked at more closely in elegant canine studies conducted by Wasserman and colleagues. Hamilton et al. (108) observed that an intraduodenal glucose infusion (8 mg/kg/min) after 150 min of moderate intensity exercise resulted in higher arterial glucose levels due to enhanced intestinal glucose absorption. However, it was also observed that net hepatic glucose uptake was unchanged after exercise, thereby suggesting that it is the increase in intestinal absorption that increases glucose delivery to the periphery after exercise, not a decrease in *the rate* of hepatic glucose uptake. These data, along with those of Rose et al. (107) and Maehlum et al. (106), suggest that the rate of hepatic glucose uptake after exercise in young males and healthy canines is not diminished; in fact, it is unchanged. Instead, it is the accelerated intestinal absorption of glucose after exercise that facilitates the escape of more glucose from the splanchnic bed, where it is metabolized in the periphery by primarily skeletal muscle.

### Hepatic Responses to Aerobic Exercise in People With T2D

Given that hepatic insulin sensitivity is known to be enhanced after exercise training in T2D, it stands to reason that impaired SGU seen in this population would also be improved as a result of lifestyle modification. However, to date, only a handful of studies have been conducted which relate to this topic. Kawamori et al. (109) studied six patients with T2D twice; once 3 h after having performed a single bout of cycle ergometer exercise (90% of measured anaerobic threshold for a total of 200 kcal) and another after having remained sedentary. In response to the exercise bout, it was reported that the percent of the ingested glucose disposed of by the splanchnic bed doubled from 23.4 to 50.5%, thereby supporting the notion that a single bout of insulin-sensitizing exercise can improve SGU in patients with T2D. In another study, but of longer duration (2 weeks), Tamura et al. (88) had two groups of non-obese (BMI of ~27 kg/m^2^) patients with T2D undergo caloric restriction resulting in weight loss, with one of the two groups also performing regular exercise (2–3 sessions per day of 30 min walking at 50–60% of VO_2_ max, 5–6 days per week). The data from these experiments show that intrahepatic lipid content was lowered by ~25–30% in both groups and that the loss was not different between them. Then, in a secondary analysis, they measured SGU in a total of six subjects, three from each group (where the data from the two groups were combined, instead of analyzing them separately). The results showed that the percent of the oral glucose taken up by the splanchnic tissues increased from 38 to 50% within the collapsed group. In a more recent study, Gregory et al. (50) studied the effect of 15 weeks of aerobic exercise training without weight loss (70% VO_2_ max, 4–5 days/week, 50 min/day) on SGU in patients with T2D. In addition to observing that insulin-mediated suppression of EGP was improved in the same group of patients after exercise training, it was reported that SGU in response to a 75-gram oral glucose load decreased from 23 grams to 9 grams. Of interest, however, was that the increment in muscle glucose uptake after exercise (22 grams), more than made up for the 14-gram reduction in SGU, thereby leading to a net improvement in whole body glucose metabolism. At this time, the limited number of studies on the topic of exercise and SGU makes it difficult to draw definitive conclusions. However, discrepancies in results may be attributable to the exercise performed (e.g., mode, duration, intensity, etc.), and/or weight loss effects, thereby highlighting the need for future studies to better understand the effect of exercise training on SGU.

In summary, while there are a number of comprehensive studies that have examined the impact of exercise on oral glucose disposition in healthy human subjects and animals, we know relatively little about the responses of patients with T2D. At the current time, it appears that weight loss and/or reduced IHL may improve diminished SGU in this population, whereas exercise training in the absence of weight loss has not been shown to increase SGU in healthy subjects or patients with T2D. Given the liver's prominence in whole-body glucose tolerance and the known dysregulation of this process in T2D, more attention should be placed on discovering the way in which exercise training impacts post-prandial hepatic glucose metabolism. Most notably, it would be of particular benefit to know the individual and interactive effects of exercise training and weight loss on SGU. Thereafter, the study of variables such as exercise intensity and resistance training should be considered. The low number of relevant studies in the field at this time also contributes to the heterogeneity of responses, making it difficult to make side by side comparisons. Adding to this heterogeneity is the considerable between-study methodological differences (e.g., weight loss, exercise intensity, training duration, low sample size, etc.), which also contributes to differing responses. Moving forward, it will be important to minimize these methodological differences until the key tenets of SGU that are impacted by exercise training in T2D patients are distilled. Such knowledge can then be used to optimize individual treatment strategies for these people.

## Conclusion

Nearly 40 years ago, it was shown for the first time that impaired hepatic glucose metabolism in patients with T2D can be improved by regular exercise training. Since that time, our knowledge about how this is achieved has greatly expanded from studies ranging from rodents to the human. Despite these developments, fundamental questions remain about how exercise impacts liver glucose metabolism, such as the interaction between lowered IHL and hepatic responses to insulin, the way in which the antagonistic relationship between insulin and glucagon on hepatic glucose metabolism is affected, hepatic responses to different exercise stimuli (e.g., resistance training, intensity-dependent effects) and the nature of the benefit of exercise training on SGU. Furthering our knowledge in this field could lead to the emergence of novel treatment strategies and reduce the negative impact of debilitating vascular complications on the lives of patients with T2D.

## Author Contributions

SW, MY, and JW wrote and edited the manuscript. RC prepared the figures and provided feedback on the manuscript. All authors contributed to the article and approved the submitted version.

## Conflict of Interest

The authors declare that the research was conducted in the absence of any commercial or financial relationships that could be construed as a potential conflict of interest.
